# Epigenetic age acceleration in surviving versus deceased COVID-19 patients with acute respiratory distress syndrome following hospitalization

**DOI:** 10.1186/s13148-023-01597-4

**Published:** 2023-11-28

**Authors:** Yosra Bejaoui, Fathima Humaira Amanullah, Mohamad Saad, Sara Taleb, Martina Bradic, Andre Megarbane, Ali Ait Hssain, Charbel Abi Khalil, Nady El Hajj

**Affiliations:** 1 College of Health and Life Sciences, Hamad Bin Khalifa University, Qatar Foundation, Education City, Doha, Qatar; 2 Qatar Computing Research Institute, Hamad Bin Khalifa University, Qatar Foundation, Doha, Qatar; 3 Proteomics Core Lab, Weill Cornell Medicine-Qatar, Doha, Qatar; 4 Department of Genetic Medicine, Weill Cornell Medicine, New York, USA; 5 Marie-Josee and Henry R. Kravis Center for Molecular Oncology, Memorial Sloan Kettering Cancer Center, New York, NY USA; 6 Gilbert and Rose-Mary Chagoury School of Medicine, Lebanese American Univeristy, Beirut, Lebanon; 7 Medical Intensive Care Unit, Hamad Medical Corporation, Doha, Qatar; 8 Epigenetics Cardiovascular Lab, Weill Cornell Medicine-Qatar, Doha, Qatar; 9 Joan and Sanford I. Weill Department of Medicine, Weill Cornell Medicine, New York, USA; 10 College of Science and Engineering, Hamad Bin Khalifa University, Qatar Foundation, Doha, Qatar

**Keywords:** COVID-19, Epigenetic clocks, Epigenetic age acceleration, SARS-CoV-2

## Abstract

**Background:**

Aging has been reported as a major risk factor for severe symptoms and higher mortality rates in COVID-19 patients. Molecular hallmarks such as epigenetic alterations and telomere attenuation reflect the biological process of aging. Epigenetic clocks have been shown to be valuable tools for measuring biological age in various tissues and samples. As such, these epigenetic clocks can determine accelerated biological aging and time-to-mortality across various tissues. Previous reports have shown accelerated biological aging and telomere attrition acceleration following SARS-CoV-2 infection. However, the effect of accelerated epigenetic aging on outcome (death/recovery) in COVID-19 patients with acute respiratory distress syndrome (ARDS) has not been well investigated.

**Results:**

In this study, we measured DNA methylation age and telomere attrition in 87 severe COVID-19 cases with ARDS under mechanical ventilation. Furthermore, we compared dynamic changes in epigenetic aging across multiple time points until recovery or death. Epigenetic age was measured using the Horvath, Hannum, DNAm skin and blood, GrimAge, and PhenoAge clocks, whereas telomere length was calculated using the surrogate marker DNAmTL. Our analysis revealed significant accelerated epigenetic aging but no telomere attrition acceleration in severe COVID-19 cases. In addition, we observed epigenetic age deceleration at inclusion versus end of follow-up in recovered but not in deceased COVID-19 cases using certain clocks. When comparing dynamic changes in epigenetic age acceleration (EAA), we detected higher EAA using both the Horvath and PhenoAge clocks in deceased versus recovered patients. The DNAmTL measurements revealed telomere attrition acceleration in deceased COVID-19 patients between inclusion and end of follow-up and a significant change in dynamic telomere attrition acceleration when comparing patients who recovered versus those who died.

**Conclusions:**

EAA and telomere attrition acceleration were associated with treatment outcomes in hospitalized COVID-19 patients with ARDS. A better understanding of the long-term effects of EAA in COVID-19 patients and how they might contribute to long COVID symptoms in recovered individuals is urgently needed.

**Supplementary Information:**

The online version contains supplementary material available at 10.1186/s13148-023-01597-4.

## Background

The global outbreak of COVID-19 resulted in a significant public health crisis with wide-ranging implications. According to the World Health Organization, over 767 million confirmed cases and 7 million deaths have been attributed to COVID-19 as of July 2023 (https://covid19.who.int). COVID-19 is caused by an enveloped single-stranded positive RNA virus known as severe acute respiratory coronavirus 2 (SARS-CoV-2), which first emerged in Wuhan City, China, in late 2019 [1]. The main causes of death in patients with COVID-19 are respiratory failure and multiorgan dysfunction due to impaired immune response and uncontrolled inflammatory processes [2]. Patients with severe types of COVID-19 frequently experience respiratory failure and may often require mechanical ventilation [3]. Although the major signs and symptoms of COVID-19 are currently well known, there is increasing evidence that the virus may have long-term effects and may impact many different aspects of human health. Those ongoing health problems following initial COVID-19 infection are commonly referred to as Long COVID or Post-COVID Conditions [4].

Chronological age is one of the well-established prognostic factors in patients with COVID-19 independent of other age-related comorbidities such as diabetes and cardiovascular diseases [5]. COVID-19 poses a disproportionate threat to older adults due to the increased risk of disease severity, mortality rates, and long-term consequences [6] in contrast to infants and children who often exhibit milder clinical symptoms. Apart of aging, SARS-CoV-2 genomic mutations in both untranslated regions and gene regions were also reported to be associated with increased risk for severe symptoms in COVID-19 patients [7]. Aging is a complex biological process characterized by a progressive decline in physiological function and increased disease susceptibility. Aging is defined by specific hallmarks such as genomic instability, loss of proteases, stem cell exhaustion, telomere attrition, and epigenetic alterations [8]. The most studied type of epigenetic alteration is DNA methylation, which is an addition of a methyl group to the 5^th^ carbon position of the cytosine ring to form 5-methylcytosine. This modification mainly occurs within the context of a CpG dinucleotide and is known to regulate gene expression [9]. DNA methylation signatures are known to be impacted by environmental exposures and are strongly correlated with aging in multiple tissues [10–12].

DNAm age often referred to as epigenetic age is a measure of biological age based on DNA methylation of specific CpG sites that reflect environmental exposures and disease risks [13]. Epigenetic age acceleration has been reported to be associated with multiple diseases including cancer, diabetes, Alzheimer’s disease, HIV infection, and certain progeroid syndromes [11, 13–16]. Several epigenetic clocks have been developed to estimate epigenetic age [17], such as the pan-tissue Horvath clock based on 353 CpGs [18], the PhenoAge clock based on 513 CpGs [19], and the GrimAge based on 1030 CpGs associated with physiological and stress risk factors [20]. In addition, DNA methylation biomarkers can be used to estimate telomere length, which is another hallmark of aging. The DNAm telomere length estimator (DNAmTL) is based on methylation measurement of 140 CpG sites [21]. Recent research has shown a potential link between COVID-19 and accelerated aging, as demonstrated by changes in the epigenetic age [22]. Given the severity and global impact of the COVID-19 pandemic, investigating the potential influence of SARS-CoV-2 infection on accelerated biological aging is of significant public health relevance. Studies have reported intriguing findings indicating that COVID-19 patients exhibit accelerated epigenetic aging compared to healthy individuals of similar chronological age [22]. Furthermore, a significant acceleration of telomere attrition was observed comparing healthy individuals versus COVID-19 patients. Severe COVID-19 infection often leads to respiratory failure requiring prolonged mechanical ventilation. A recent study by Cao et al*.* reported accelerated epigenetic aging to be associated with the severity of COVID-19; however, the underlying mechanisms and the broader implications of these observations remain poorly understood [22]. Similarly, Chamberlain et al. recently investigated the association between COVID-19 severity and biological age using two publicly available datasets. A lower biological age (measured via the Hannum and PhenoAge clocks) compared to chronological age was associated with reduced odds of COVID-19 severity. The authors also observed that PhenoAge was associated with mortality in COVID-19 patients [23].

Our study aimed to comprehensively examine epigenetic age acceleration in COVID-19 patients with acute respiratory distress syndrome (ARDS) and how it relates to outcome (survival or death) following hospitalization. In addition, we studied dynamic epigenetic aging across severe COVID-19 disease phases starting from intensive care unit (ICU) admission until death or recovery. The epigenetic age acceleration (EAA) was calculated using several clocks including Horvath, Hannum, DNAm skin and blood, GrimAge, and PhenoAge clocks. Furthermore, telomere length was estimated via the surrogate marker DNAmTL to elucidate the impact of COVID-19 on telomere attrition.

## Results

### Epigenetic age acceleration in COVID-19 patients at baseline

**Fig. 1:**
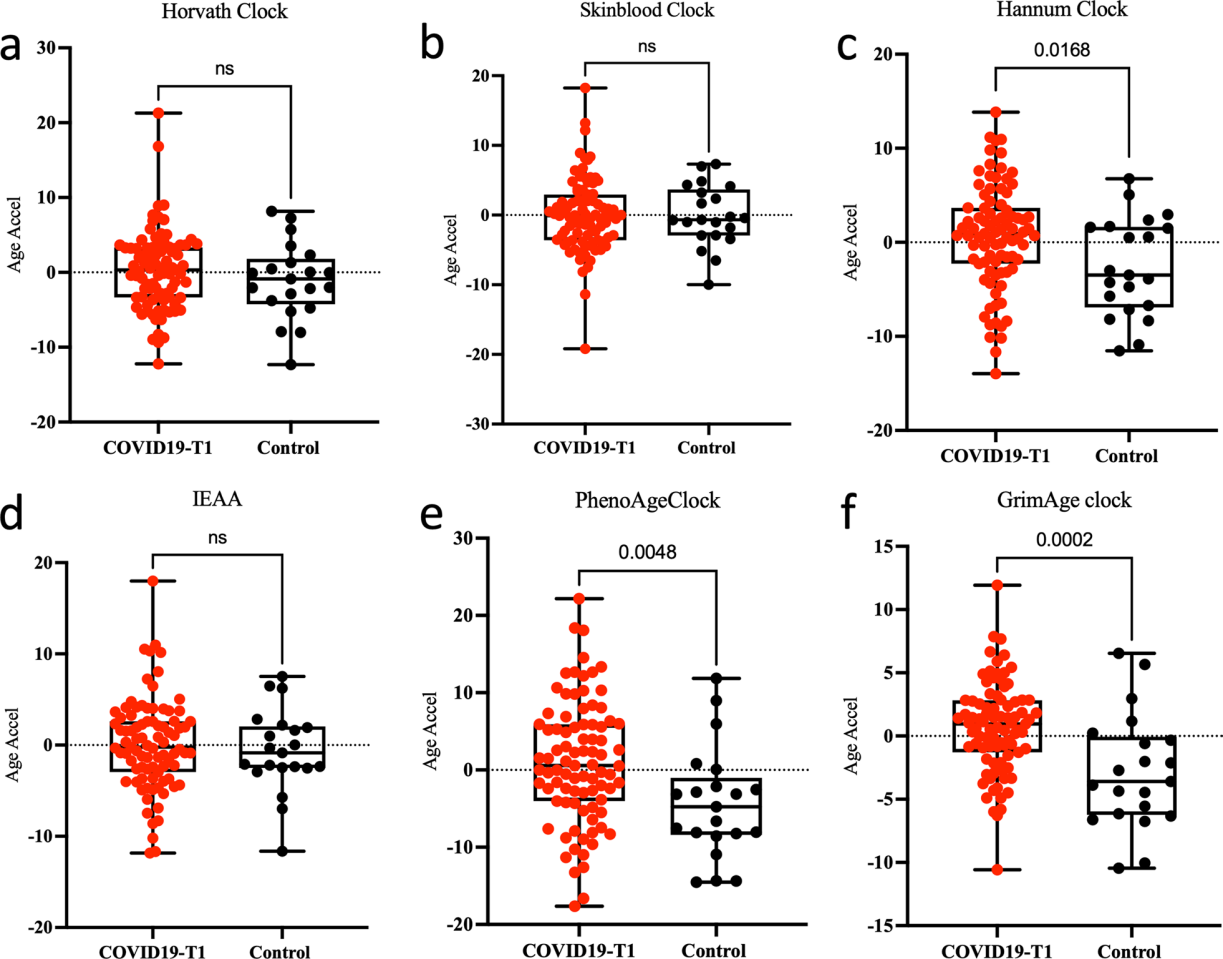
Accelerated epigenetic aging in COVID-19 patients at T1 versus controls. DNAm age acceleration measured using  six epigenetic clocks (**a**–**f**) in the peripheral blood from 21 control samples and 87 severe COVID-19-T1patients. The *y*-axis shows the epigenetic age. The *p*-value for each *t*-test is shown above the corresponding line. In the box plots, the lower and upper hinges indicate the 25th and 75th percentiles and the black line within the box marks the median value. ns: non-significant

We measured epigenetic age using five different epigenetic clocks in whole blood samples collected from 87 hospitalized COVID-19 patients with ARDS under mechanical ventilation at inclusion time-point T1 and 21 control samples. A strong positive correlation was observed when comparing epigenetic age versus chronological age using the different clocks including Horvath (*r* = 0.87, *p* = 2.5e−34), SkinBlood (*r* = 0.92, *p* = 6.3e−45), Hannum (*r* = 0.88, *p* = 4.7e−36), PhenoAge (*r* = 0.83, *p* = 1.2e−28), and GrimAge (*r* = 0.93, *p* = 7e−48) (Additional file 1). Our analysis on Epigenetic Age Acceleration (EAA) in COVID-19 patients revealed significant acceleration when compared to control samples in three different epigenetic clocks: Hannum clock (*p* = 0.0168), PhenoAge clock (*p* = 0.0048), and GrimAge clock (*p* = 0.002) after adjusting for BMI and gender (Fig. 1).

### Epigenetic age acceleration in deceased and recovered critically ill COVID-19 patients

In total, 78% of the COVID-19 patients (*N* = 68) survived and were discharged from ICU at different time points. Patients who died were on average older compared to those who recovered (*p* < 0.001) (Table 1).

**Table 1:** Clinical characteristics of surviving and deceased COVID-19 patients following hospitalization

	COVID-19 Survived (*N* = 68)	COVID-19 Died (*N* = 19)	Controls (*N* = 21)
Age	47 (41–53.5)	58 (52.5–63.5)	40 (34–45)
Epigenetic age			
DNAmAge	52.4 (46.7–60.1)	57.7 (54.5–63)	43.5 (40.4–52.1)
DNAmAgeHannum	38.2 (33.3–45.1)	44.7 (39.9–51.4)	25.6 (23.5–33.3)
DNAmPhenoAge	40.6 (34.3–50.1)	50.6 (44–58.8)	27.6 (20.6–38.1)
DNAmAgeSkinBloodClock	46.5 (40.7–56.2)	54.4 (50.3–61)	38.6 (35.1–46.2)
DNAmGrimAge	55.5 (50.8–61.2)	63.8 (60.3–68.7)	46 (39.4–52.7)
Gender (male)	63 (93%)	19 (100%)	21 (100%)
BMI (kg/m^2^)	27.4 (25.3–31.4)	25.7 (24.4–29.9)	28.7 (26–30.1)
Duration of MV (days)	7 (4–13.5)	20 (14.5–26.5)	-
ICU LoS (days)	14 (9.7–26.2)	24 (15–30.5)	-
Hospital LoS (days)	30 (22–46)	25 (17–35)	-
ECMO	7 (10%)	4 (21%)	
Nosocomial infections	35 (51.5%)	15 (78.9%)	-
Convalescent plasma therapy	21 (30.9%)	6 (31.6%)	-
Diabetes status			
Non diabetes	34 (50%)	12 (63.2%)	–
Pre-diabetes	2 (3%)	1 (5.3%)	–
Diabetes	32 (47%)	6 (31.6%)	–
Hypertension	29 (42.9%)	9 (47.4%)	–
Coronary artery disease	4 (5.9%)	1 (5.3%)	–
Chronic kidney failure	5 (7.4%)	2 (10.5%)	–
Chronic heart failure	1 (1.5%)	0	–

Data are represented as number of cases (%) per each category for categorical variables and as median (1st–3rd quartile) for continuous variables

*ICU* Intensive care unit, *LoS* Length of stay, *MV* Mechanical ventilation. *ECMO* Extracorporeal membrane oxygenation

**Fig. 2:**
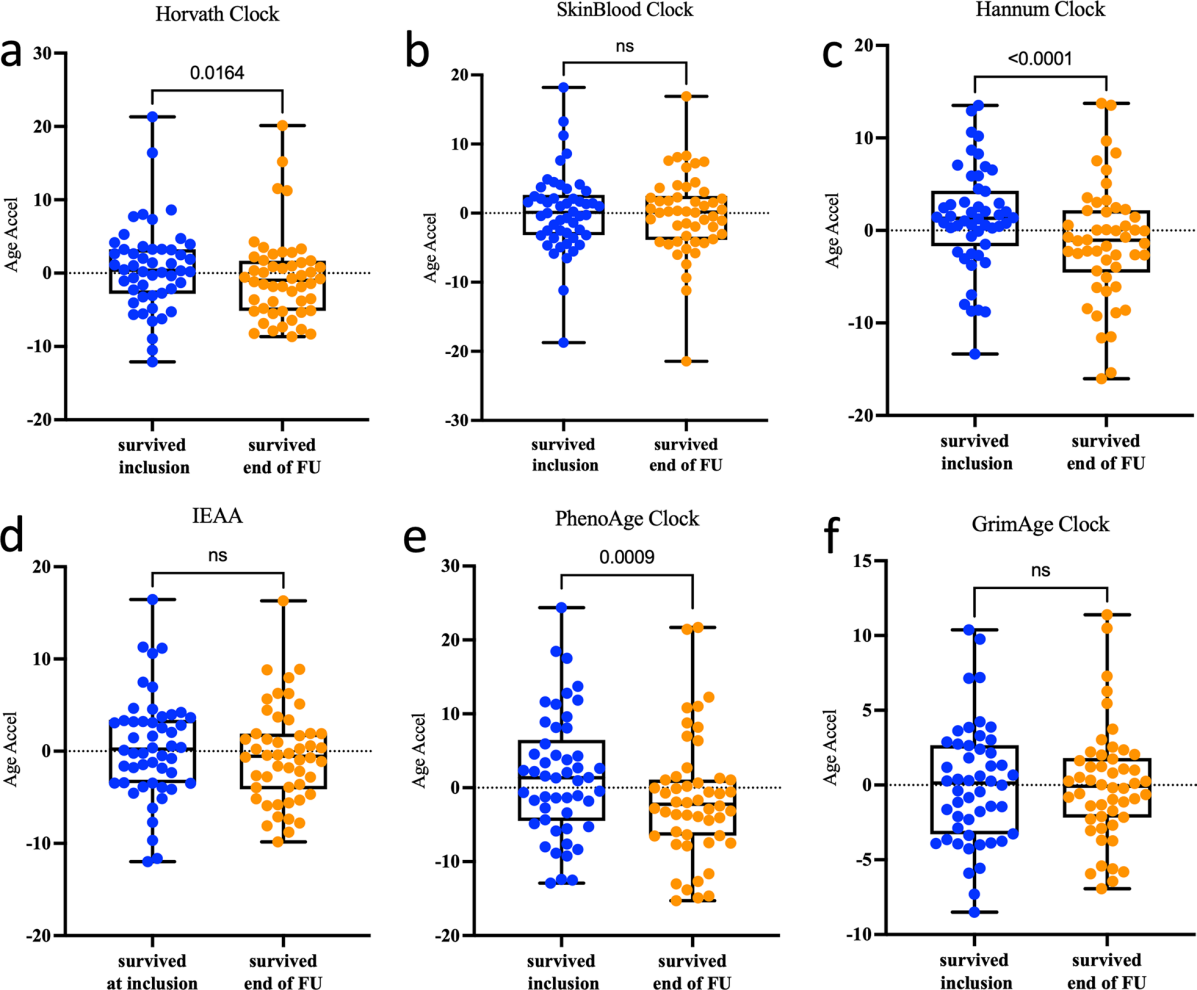
Epigenetic age acceleration at inclusion to the last recorded DNAmAge before recovery i.e., end of Follow-Up (FU). DNAm age acceleration measured via six epigenetic clocks (**a**–**f**) in the peripheral blood from 50 COVID-19-T1 survived patients. The y-axis shows the epigenetic age acceleration. The *p* value is shown above the corresponding line. In the box plots, the lower and upper hinges indicate the 25th and 75th percentiles and the black line within the box marks the median value. ns: non-significant

In addition, they had a lower BMI, and were more likely to have hypertension and chronic kidney disease, yet the difference was not significant between both groups (*p* > 0.05). First, we measured EAA in the COVID-19 patients who recovered after being admitted to the ICU. The initial analysis included 87 patients, of which 68 individuals survived and 19 died during treatment. Unfortunately, the end of the follow-up time point was missing in some of those individuals, leaving us with data from 50 COVID-19 survivors who had both time points available, along with data from 14 COVID-19 patients who died in the ICU. We examined DNAmAge at inclusion (T1) for the surviving COVID-19 patients and compared it to their last recorded DNAmAge in the ICU before recovery. Among the different epigenetic clocks used in our study, the Horvath clock (*p* = 0.0164), Hannum clock (*p* < 0.0001) and PhenoAge clock (*p* = 0.0009) revealed a significant decrease in EAA at the last recorded timepoint before recovery (Fig. 2).

Next, we measured EAA in the subset of 14 COVID-19 patients who died following ICU admission. Comparing DNAmAge at inclusion to the last recorded DNAmAge before death revealed no significant EAA using the different epigenetic clocks (Additional file 2).

**Fig. 3:**
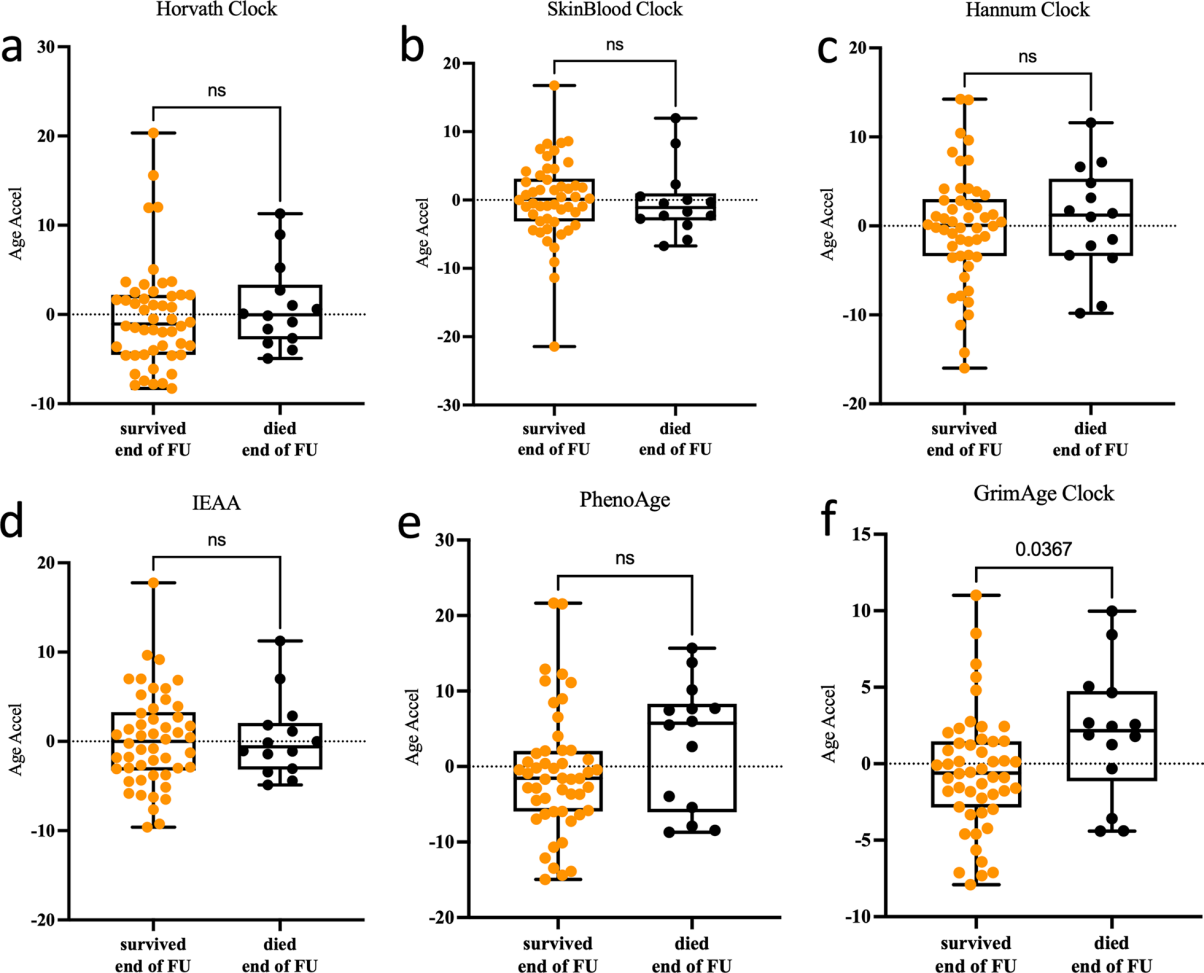
Epigenetic age acceleration in survived versus deceased COVID-19 patients at the end of follow-up (FU) timepoint. Boxplots of DNAm age acceleration measured using six epigenetic clocks (**a**–**f**) in the peripheral blood from 50 surviving versus 14 deceased COVID-19 patients at the end of follow-up. The *y*-axis denotes the epigenetic age acceleration. *p* value is shown above the corresponding line. In the box plots, the lower and upper hinges indicate the 25th and 75th percentiles and the black line within the box represents the median. ns: non-significant

We additionally compared DNAmAge in COVID-19 patients who died to those who recovered at both the baseline level and the final time point of follow-up. This analysis revealed no EAA using different epigenetic clocks apart of in the GrimAge clock (Fig. 3).

### Longitudinal epigenetic age acceleration across disease phases

**Fig. 4:**
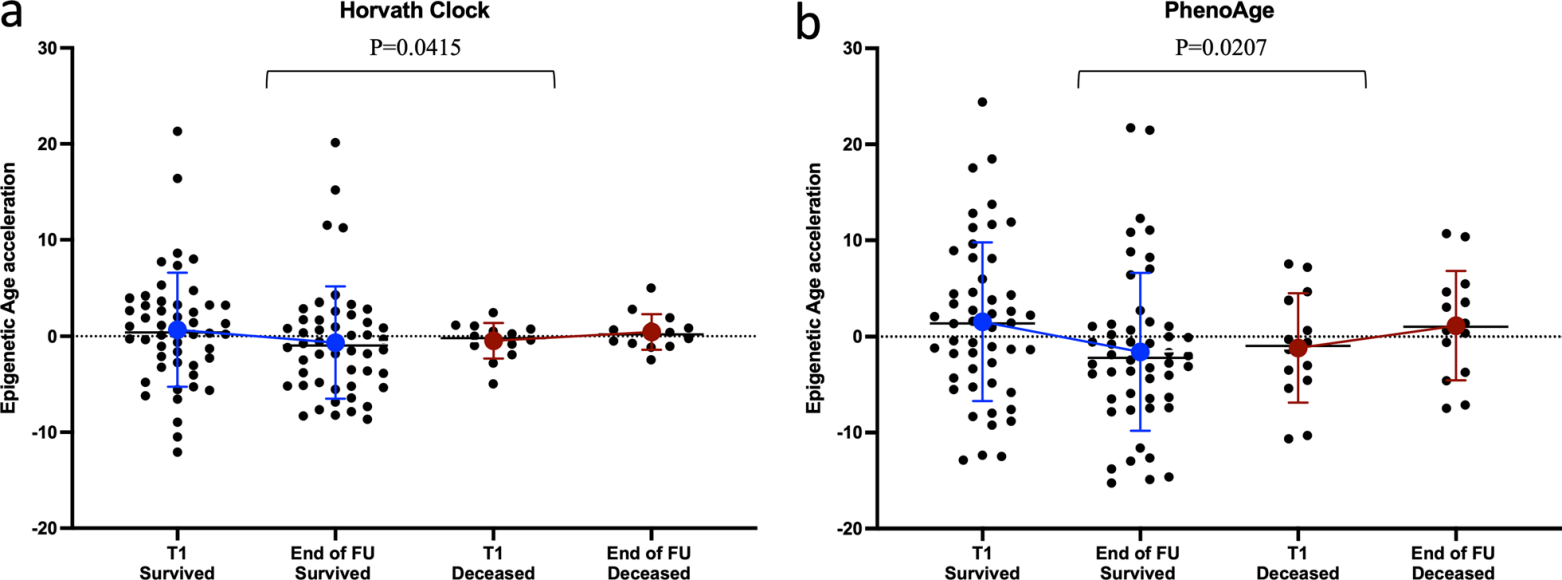
Dynamic change in epigenetic age acceleration in survived versus deceased COVID-19 patients between inclusion and the end of follow-up (FU) timepoint. Dynamic change in DNAm age acceleration measured via the **a**. Horvath and **b**. PhenoAge clocks in the peripheral blood from 50 surviving versus 14 deceased COVID-19 patients. The y-axis denotes the epigenetic age acceleration. *p* value is shown above the corresponding line and compares EAA between the end of follow-up and inclusion (EAA end of follow-up—EAA inclusion) in the survived versus deceased group. In the box plots, the lower and upper hinges indicate the 25th and 75th percentiles and the black line within the box represents the median

Here, we used a linear mixed model to examine the dynamic acceleration of epigenetic age over continuous time points (T1-T5) of 50 COVID-19 patients who survived and 14 who died. Our analysis of dynamic EAA across these different time points showed no significant difference between COVID-19 patients who survived versus those who died following hospitalization in all of the employed epigenetic clocks. We additionally compared dynamic changes in EAA by calculating the difference between EAA at the end of follow-up and at inclusion time points in both groups, which we defined as “Change in AgeAcceleration.” In this analysis, we could observe a significant increase in EAA using both the Horvath and the PhenoAge clocks (*p* = 0.0415 and 0.0207, respectively) (Fig. 4, Additional file 3).

### Telomere attrition in COVID-19 patients

**Fig. 5:**
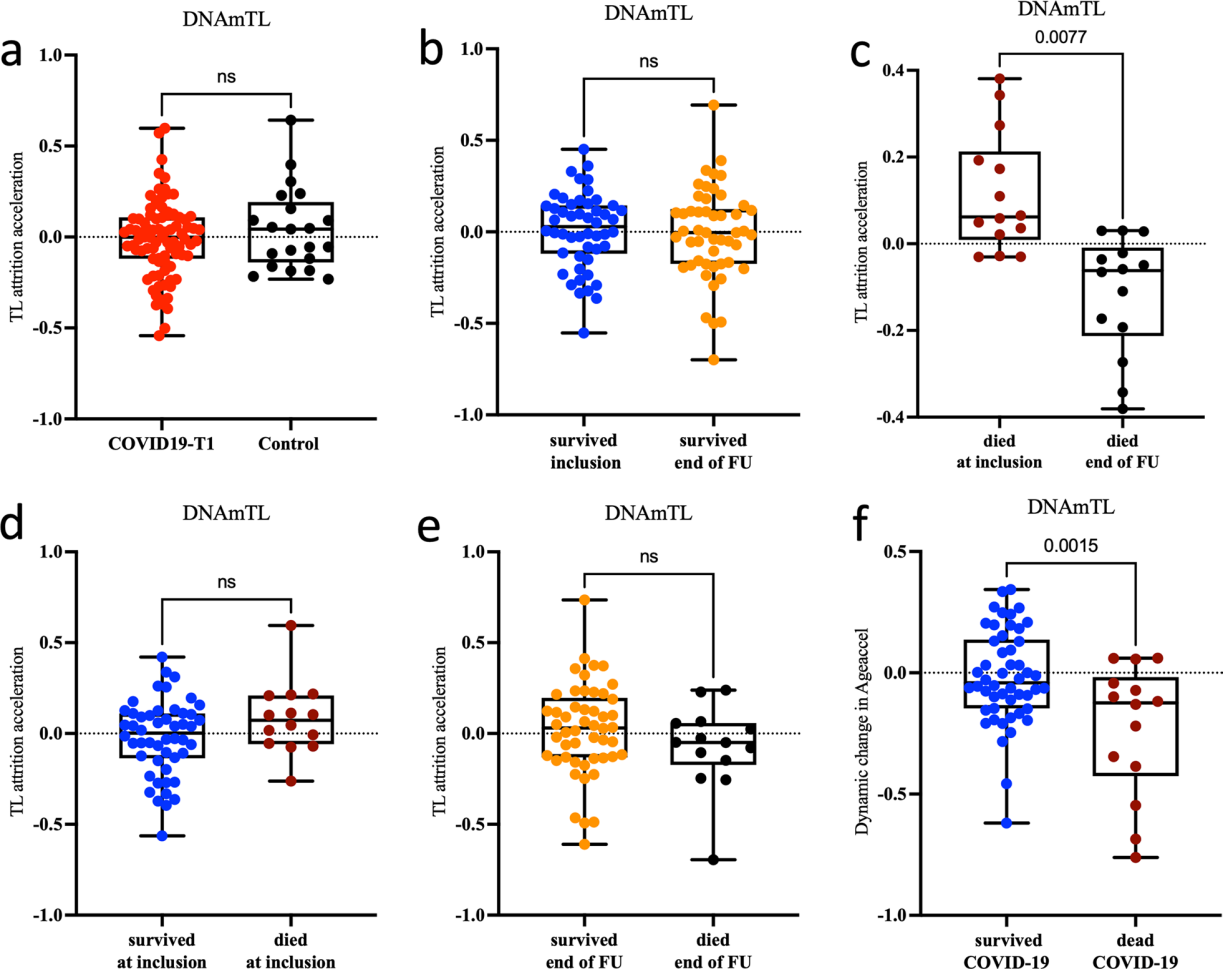
Telomere attrition in COVID-19 patients. Telomere attrition acceleration measured via the DNAm TL surrogate marker (**a**–**f**) in the peripheral blood of COVID-19 patients. Telomere attrition acceleration in **a** COVID-19 patients at baseline (T1) versus controls **b** in surviving COVID-19 patients at inclusion versus the last recorded measurement at the end of Follow-Up (FU) **c)** in deceased COVID-19 patients at inclusion versus end of FU **d** in survived versus deceased patients at T1and **e** at the end of FU. **f** Dynamic change in telomere attrition acceleration between the end of follow-up and inclusion. The y-axis denotes telomere attrition acceleration apart from panel (**f**), representing a change in telomere attrition acceleration between the end of FU and inclusion. *p* value is shown above the corresponding line. In the box plots, the lower and upper hinges indicate the 25th and 75th percentiles and the black line within the box represents the median. ns: non-significant

We calculated telomere length using the surrogate marker DNAm TL to compare accelerated telomere attrition in the studied cohort. The initial analysis of COVID-19 samples versus controls revealed no significant difference in DNAmTL attrition (Fig. 5a).

When comparing telomere attrition acceleration between inclusion and end of follow-up, we observed no changes in DNAmTL attrition in the critically ill, COVID-19 patients who recovered. However, we detected a significant DNAmTL attrition in the deceased COVID-19 patients at the end-of-follow-up (*p* = 0.0077) indicating a measurable reduction in telomere length in these individuals (Fig. 5b–e). Finally, we compared dynamic differences in telomere attrition by calculating the variable “Dynamic Change in TL attrition acceleration” after subtracting TL attrition acceleration at inclusion from the end of the follow-up. Comparing Dynamic Change in TL attrition acceleration showed a significant reduction in TL attrition acceleration in the deceased COVID-19 patients between the two time-points (*p* = 0.0015; Fig. 5f). Nevertheless, when we applied a mixed linear model over the continuous time points (T1–T5) for TL attrition acceleration, we did not observe any difference between the deceased versus surviving COVID19 patients.

## Discussion

In this longitudinal study, we performed an epigenetic age acceleration analysis in critically ill COVID-19 patients with ARDS receiving mechanical ventilation. Overall, we observed an increased EAA in critically ill COVID-19 patients compared to non-infected controls. This EAA was detected using one of the first-generation clocks (Hannum clock) as well as the second-generation clocks (PhenoAge and GrimAge) [19, 20, 24]. Our findings reveal increased EAA in individuals with severe COVID-19 symptoms and ARDS. We subsequently examined COVID-19 patients who survived and those who died as distinct groups. Among survivors, we identified a decrease in EAA using the Horvath, Hannum, and PhenoAge clocks at the last recorded timepoint before recovery. In contrast, there was no significant change in EAA between inclusion and the last recorded timepoint before death among those who died.

A handful of studies reported an effect of viral infection including HIV and SARS-CoV-2 on epigenetic aging [15, 25, 26]. Corley et al. first reported increased DNAm age measured via the second-generation GrimAge clock in severe COVID-19 cases. In contrast, Franzen et al. did not observe EAA in hospitalized COVID-19 patients with and without ARDS using four first-generation DNAm age predictors [27, 28]. More recently, Cao et al. reported accelerated epigenetic aging associated with SARS-CoV-2 infection and COVID-19 disease severity. The study used multiple epigenetic clocks including first- and second-generation clocks and looked at a large cohort of 194 patients with mild/moderate symptoms and 213 severe COVID-19 patients [22]. Their findings align with our study’s observations, particularly for certain epigenetic clocks.

Furthermore, we could observe that reversing epigenetic age (i.e., epigenetic deceleration) measured by the Horvath, Hannum, and PhenoAge clock is associated with recovery in severe COVID-19 patients following hospitalization. In contrast, patients who died after ICU admission did not show any differences in epigenetic aging at inclusion to the last recorded DNAm age before death. This indicates that COVID-19 alters epigenetic aging and decelerating this EAA would lead to recovery following ICU admission. Recently, Poganik et al. reported transient changes in epigenetic aging in patients with severe COVID-19 and during surgery, where patients exhibited an increase in biological aging following exposure to stress that was later reversed after recovery [29]. This aligns with the findings from our study where EAA was reversed in patients that recovered following hospitalization. Similarly, the previously mentioned study by Cao et al. analyzed dynamic changes in EAA during multiple disease phases, where the authors observed DNAm age acceleration at the initial phase of infection to be partly reversed at the later convalescence phase [22]. One crucial question is whether the observed EAA is causally related to disease risk and severity or is a consequence of SARS-CoV-2 infection. In this context, a single Mendelian randomization study looked at the causal relationship between COVID-19 and epigenetic aging. However no causal association was observed between epigenetic age and COVID-19 susceptibility [30]. In addition, a recent study reported several significant differentially methylated sites associated with aging in COVID-19 patients including the *ELOVL2* gene which accounts for several CpG sites in the Hannum epigenetic clock [31].

Interestingly, our study observed that COVID-19 patients who died following mechanical ventilation exhibited significant telomere shortening at the last recorded timepoint before death when compared to the first timepoint at ICU admission. This suggests that critically ill patients with telomere attrition are more likely to die during hospitalization. Similarly, dynamic change in TL attrition acceleration between the end of follow-up and inclusion revealed significant differences between survived versus deceased patients. Despite conflicting data, several studies including a meta-analyses showed an increased risk for all-cause mortality to be associated with shorter telomeres in the general population [32–34]. It is important to mention that we could not identify differences in telomere attrition between COVID-19 patients and controls, which is in contrary to other studies reporting telomere shortening following SARS-CoV-2 infection [22, 35]. Similar to our study, Cao et al. used the DNAmTL surrogate marker to measure telomere length [22]. In contrast, Mongelli et al. employed a qPCR-based assay for absolute telomere length quantification [35]. The output of DNAmTL is known to exhibit a moderately strong association with telomere length measured using qPCR or southern blotting in the blood [21]. However, our findings align with the study of Franzen et al*.* where lymphocytes from severe COVID-19 patients did not show a significant acceleration of telomere attrition [28].

Regarding dynamic changes in EAA during disease phases, the study by Cao et al. looked at a previously published dataset of only six COVID-19 cases and six uninfected controls [22, 36]. To our knowledge, our study is the first to look at EAA following a longitudinal follow-up of several severe COVID-19 cases with ARDS and to determine the relationship between EAA/Telomere attrition and outcome (survival or death). Nevertheless, one of the main limitations of this study is the lower number of non-COVID controls (*N* = 21) and the limited number of studied samples who died following ICU admission. Only 14 deceased COVID-19 individuals longitudinally followed during hospitalization were included in this study. In addition, we could only determine an association between accelerated epigenetic aging and COVID-19. However, understanding the causal relationship between them was outside the scope of the current study.

## Conclusion

In conclusion, we demonstrate that severe COVID-19 is associated with a significant increase in DNA methylation age but not DNAm telomere attrition. In addition, we could also detect an association between EAA and recovery/death following hospitalization in COVID-19 patients. Similarly, an association between DNAm telomere attrition and clinical outcome was also observed. Future studies are required to explore whether epigenetic age acceleration is causally linked to disease severity and clinical outcome following hospitalization, along with the underlying biological mechanisms behind this association. Furthermore, understanding the long-term effects and consequences of accelerated aging in recovered COVID-19 patients is essential and urgently needed.

## Methods

### Ethical approval

The study is part of the “Immune Profiling of COVID-19 Patients Admitted to ICU study (IMPROVISE) (clinicaltrial.gov identifier NCT0447313). The Institutional Review Board at Hamad Medical Corporation (HMC) and Weill-Cornell approved the study with record numbers MRC-05-007 and 20-00012. All research was conducted in accordance with the ethical principles of the Declaration of Helsinki. All participants enrolled in this study or their legal guardians signed a consent form.

### Participants

In total, this study included 100 critically ill COVID-19 patients with acute respiratory distress syndrome (ARDS) who received mechanical ventilation in the intensive care unit (WHO clinical progression scale 7–9) [37], and 24 non-COVID controls from HMC blood donor unit. The patients and controls were of similar ethnicity. Our previous study by Bradic et al. provides detailed inclusion and exclusion criteria for the enrolled individuals [38]. Following their admission to the intensive care unit (ICU) (Time point 1: T1), COVID-19 patients were monitored at four different time points, including days 7 (T2), 14 (T3), 21 (T4), and 60 (T5). The follow-up was carried out in accordance with the guidelines of the WHO Working Group on the Common Outcome Measure Set for the COVID-19 Clinical Research [37]. Blood samples were collected at each time point unless the patients have died or recovered at the end of the follow-up. Once patients recovered, they were discharged from the ICU and therefore not included in any further analysis. Recovery was defined as per WHO clinical criteria of less or equal to 5 on the scale of clinical progression, discontinuing mechanical ventilation, and discharge from the ICU [37].

### Methylation array data processing

Samples were processed on the Illumina Infinium MethylationEPIC Beadchip (EPIC array) which interrogates > 850,000 CpG sites across the human genome including extensive coverage of genes, promoters, CpG Islands, and enhancers. Raw IDAT files for a total of 288 samples were obtained and processed further [38]. The RnBeads package [39] was used for quality control, and noob data normalization was performed using the minfi package [40]. Following data filtration, 13 COVID-19 patients were excluded from further analysis since their samples failed to meet quality criteria. Additionally, three healthy donor samples were excluded. The subsequent analysis was conducted on a total of 87 COVID-19 patients and 21 healthy donors. After data normalization, the methylation β value for each CpG site was extracted and used as input to calculate the epigenetic age using various clocks.

### Epigenetic age calculation and DNAmTL estimation

DNAm age was estimated using various epigenetic clocks such as the Horvath pan-tissue [18], PhenoAge [19], GrimAge [20], and SkinandBlood [41] clocks via the web-based epigenetic clock calculator (https://dnamage.clockfoundation.org/). In addition, intrinsic epigenetic age acceleration (IEAA) was measured since it reflects epigenetic age independently of age-related changes in blood composition [42]. Epigenetic age acceleration (EAA) is defined as the deviation between epigenetic age and chronological age. EAA was calculated based on the residuals from regressing DNAm age on chronological age after correcting for BMI and gender. Furthermore, DNAmTL [21] was estimated where the deviation between DNAm TL and chronological age is defined as DNAm TL attrition acceleration. This measurement was calculated by adjusting for BMI and gender as covariates. All statistical analyses were performed using RStudio version (2023.3.1) and Prism Software (version 9.51). The *R* scripts used in this analysis are provided by the clock foundation team at the following link: https://dnamage.clockfoundation.org.

### Statistical analysis

The correlations between DNAm age, DNAm TL, and chronological age of the samples were evaluated using Pearson correlation (*R*). To compare samples within the same group (individuals who survived COVID-19 or individuals who died from COVID-19) at two different time points (T1 and end of follow-up), a paired *t*-test was conducted. Additionally, an unpaired *t*-test was employed to compare COVID-19 samples to control samples, and to compare individuals who survived versus those who died at the inclusion or at the end of the follow-up period. A dynamic age acceleration linear mixed model was conducted to assess changes in epigenetic age in relation to survival status. The dependent variable in the linear mixed model was the epigenetic age and the independent variable was the survival outcome (survival or death). Subsequently, statistical significance was evaluated using a chi-square test. All statistical analyses were performed using RStudio version (2023.3.1) and Prism Software (version 9.51). *p* values < 0.05 were considered statistically significant.

### Supplementary Information

**Additional file 1.** Correlation of chronological age with DNA methylation age using Horvath, Hannum, SkinandBlood, PhenoAge, and GrimAge clocks and the DNA methylation-based telomere length (TL) estimator.

**Additional file 2.** Distribution of DNAm age acceleration in six epigenetic clocks (**a**–**f**) in the peripheral blood from 14 COVID-19 patients at inclusion versus end of follow-up. The *y*-axis shows the epigenetic age acceleration. The *p* value is shown above the corresponding line. In the box plots, the lower and upper hinges indicate the 25th and 75th percentiles and the black line within the box marks the median. ns: non-significant.

**Additional file 3.** Dynamic change in DNAm age acceleration in six epigenetic clocks (**a**–**f**) in the peripheral blood from 50 surviving versus 14 deceased COVID-19 patients. The *y*-axis denotes the difference in epigenetic age acceleration between the end of follow-up and inclusion (EAA end of follow-up—EAA inclusion). *p* value is shown above the corresponding line. In the box plots, the lower and upper hinges indicate the 25th and 75th percentiles and the black line within the box represents the median. ns: non-significant.

## Data Availability

The datasets used in the current study are available from the corresponding authors upon request.
