# Supplementary figures and images for: Epigenetic age acceleration in surviving versus deceased COVID-19 patients with acute respiratory distress syndrome following hospitalization
Source: Clin Epigenetics. 2023 Nov 28;15:186. doi: 10.1186/s13148-023-01597-4 (PMC10685564; doi:10.1186/s13148-023-01597-4)

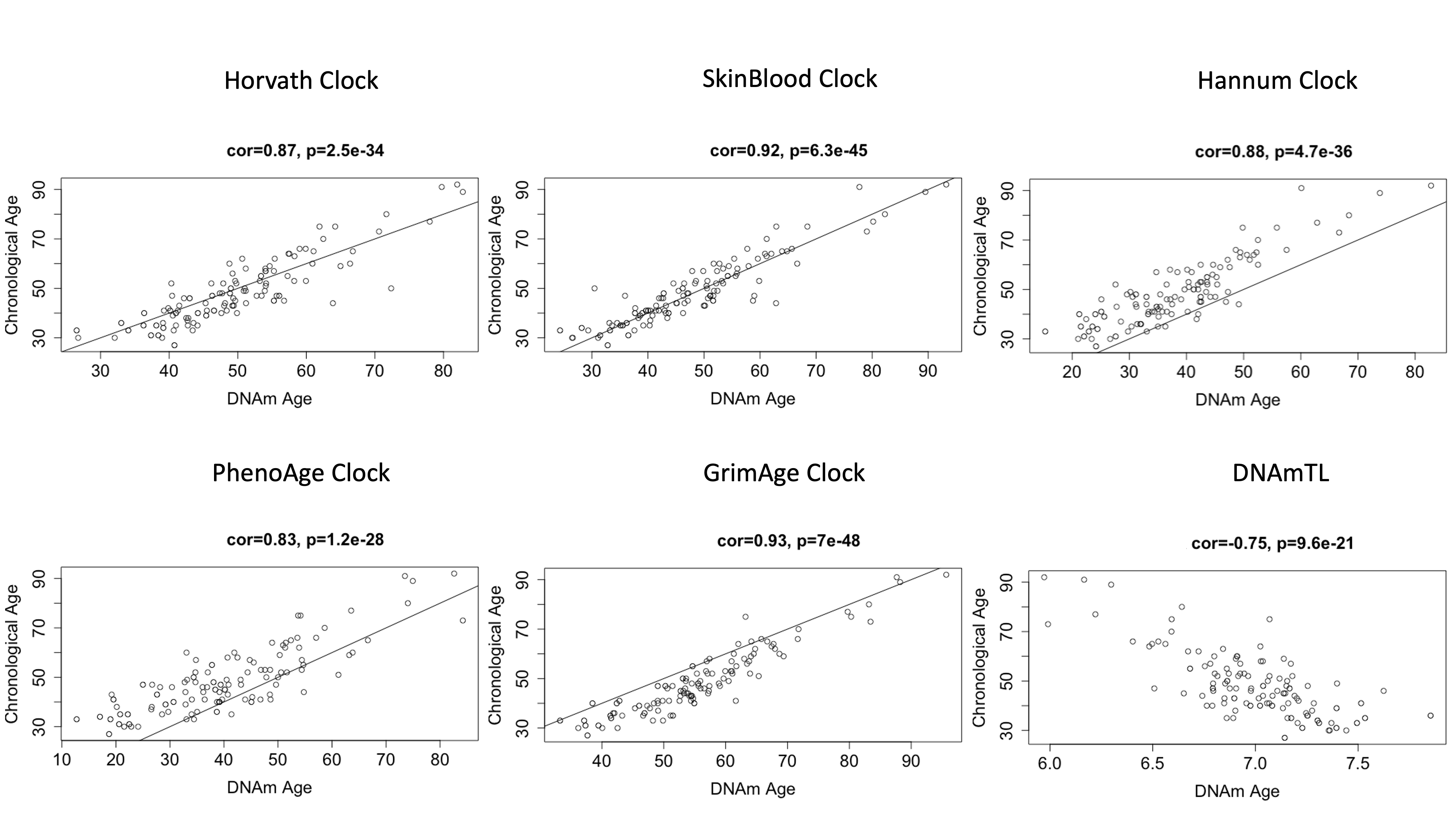

Supplement: Supplementary file 1 — Additional file 1. Correlation of chronological age with DNA methylation age using Horvath, Hannum, SkinandBlood, PhenoAge, and GrimAge clocks and the DNA methylation-based telomere length (TL) estimator. [file 13148_2023_1597_MOESM1_ESM.zip › Additional File 1.tiff]

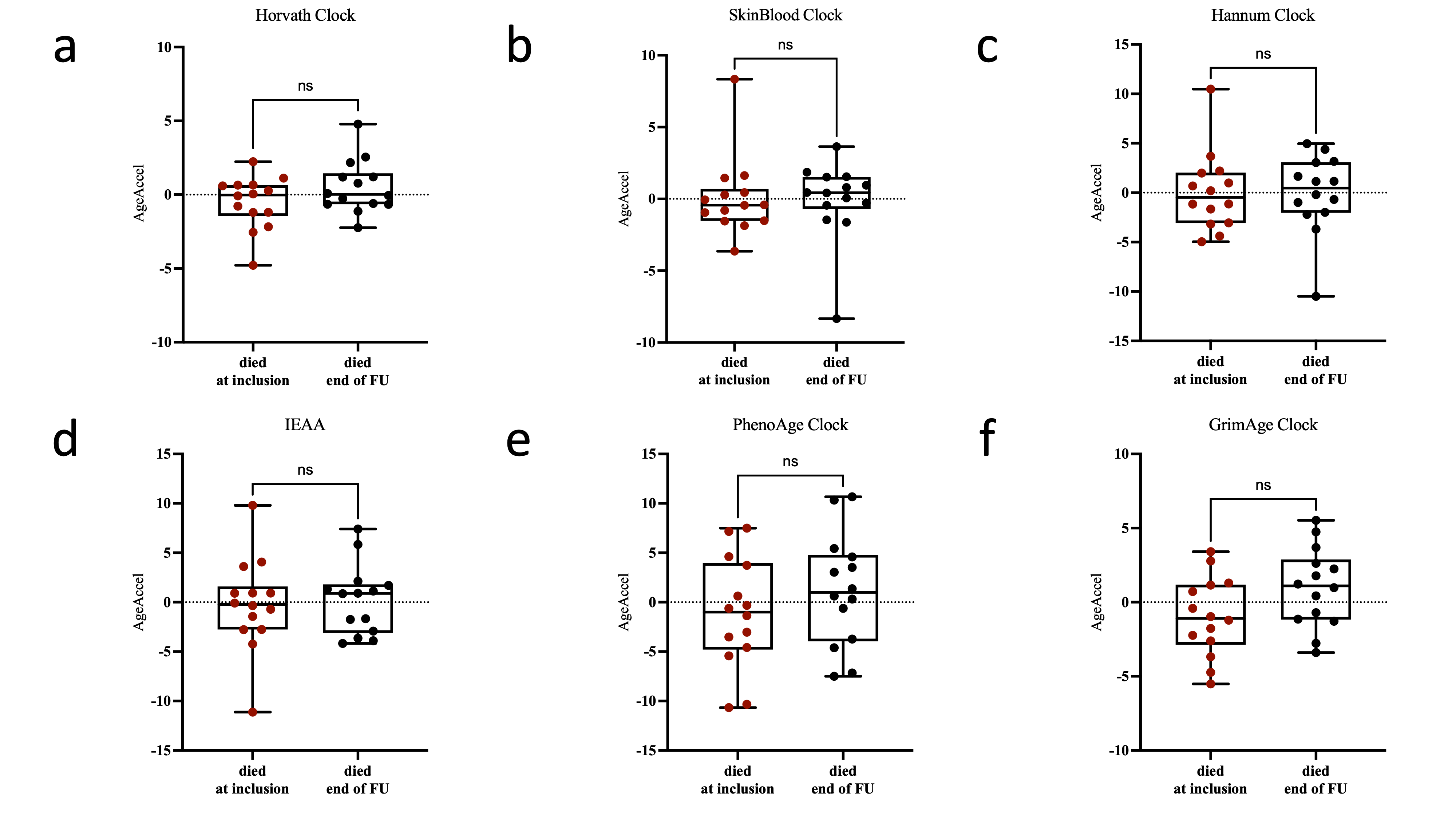

Supplement: Supplementary file 2 — Additional file 2. Distribution of DNAm age acceleration in six epigenetic clocks (a–f) in the peripheral blood from 14 COVID-19 patients at inclusion versus end of follow-up. The y-axis shows the epigenetic age acceleration. The p value is shown above the corresponding line. In the box plots, the lower and upper hinges indicate the 25th and 75th percentiles and the black line within the box marks the median. ns: non-significant. [file 13148_2023_1597_MOESM2_ESM.zip › Additiona File 2.tiff]

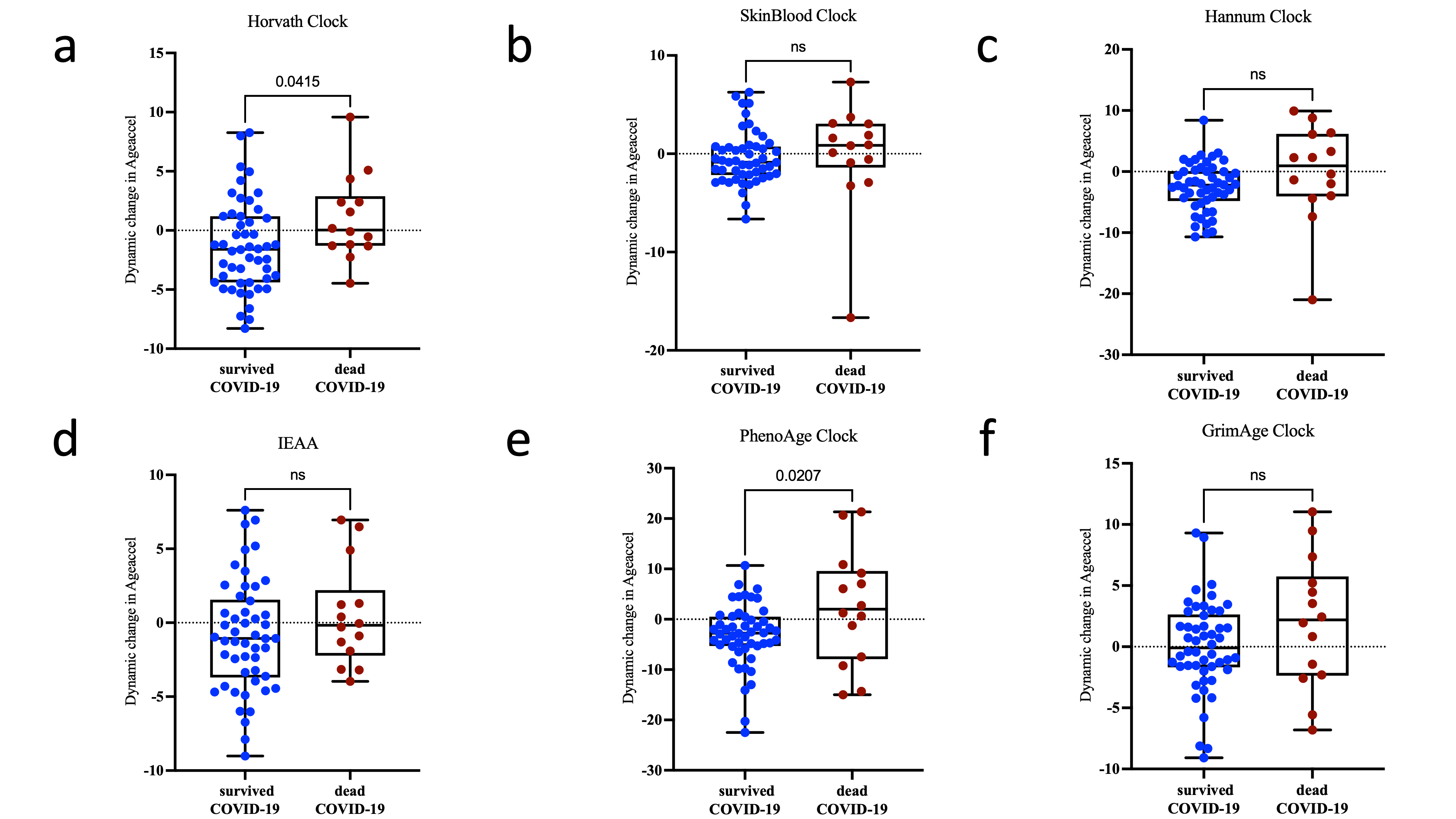

Supplement: Supplementary file 3 — Additional file 3. Dynamic change in DNAm age acceleration in six epigenetic clocks (a–f) in the peripheral blood from 50 surviving versus 14 deceased COVID-19 patients. The y-axis denotes the difference in epigenetic age acceleration between the end of follow-up and inclusion (EAA end of follow-up—EAA inclusion). p value is shown above the corresponding line. In the box plots, the lower and upper hinges indicate the 25th and 75th percentiles and the black line within the box represents the median. ns: non-significant. [file 13148_2023_1597_MOESM3_ESM.zip › Additional File 3.tiff]
